# Umbilical Cord Mesenchymal Stem Cell-Derived Nanovesicles Potentiate the Bone-Formation Efficacy of Bone Morphogenetic Protein 2

**DOI:** 10.3390/ijms21176425

**Published:** 2020-09-03

**Authors:** Songhyun Lim, Hao-Zhen Lyu, Ju-Ro Lee, Shi Huan Han, Jae Hyup Lee, Byung-Soo Kim

**Affiliations:** 1 School of Chemical and Biological Engineering, Seoul National University, Seoul 08826, Korea; petitrsh@snu.ac.kr (S.L.); ljr0518@snu.ac.kr (J.-R.L.); 2 Department of Orthopedic Surgery, College of Medicine, SMG-SNU Boramae Medical Center, Seoul National University, Seoul 07061, Korea; spinedrlyu@snu.ac.kr (H.-Z.L.); hansehwan@snu.ac.kr (S.H.H.); 3 Institute of Medical and Biological Engineering, Medical Research Center, Seoul National University, Seoul 07061, Korea; 4 Institute of Chemical Processes, Institute of Engineering Research, BioMAX, Seoul National University, Seoul 08826, Korea

**Keywords:** recombinant human bone morphogenetic protein 2, umbilical cord mesenchymal stem cell-derived nanovesicles, bone formation, angiogenesis, osteogenesis

## Abstract

Recombinant human bone morphogenetic protein 2 (rhBMP-2) is one of the most potent osteogenic factors used to treat bone loss. However, at higher doses, rhBMP-2 does not necessarily increase bone formation but rather increases the incidence of adverse side effects. Here, we investigated whether umbilical cord mesenchymal stem cell (UCMSC)-derived nanovesicles (NVs) further increase the in vivo bone formation at high doses of rhBMP-2. In the presence of UCMSC-derived NVs, proliferation, migration, and tube formation of human umbilical vein endothelial cells were stimulated in vitro. Furthermore, migration and osteogenesis of human bone marrow-derived mesenchymal stem cells were stimulated. To examine the efficacy of UCMSC-derived NVs on in vivo bone formation, collagen sponges soaked with rhBMP-2 and UCMSC-derived NVs were used in athymic nude mice with calvarial defects. At a high rhBMP-2 dosage (500 ng/mL), UCMSC-derived NVs significantly promoted bone formation in calvarial defects; however, the UCMSC-derived NVs alone did not induce in vivo bone formation. Our results indicate that UCMSC-derived NVs can potentiate the bone formation efficacy of rhBMP-2 at a high dosage.

## 1. Introduction

Bone loss occurs via trauma and surgical procedures [1]. If the bone defect is too large to spontaneously heal, bone regeneration needs to promoted to repair the defect. Bone morphogenetic proteins are one of the most effective osteogenic factors used to treat bone loss [2,3]. Specifically, recombinant human bone morphogenetic protein 2 (rhBMP-2) is a representative osteogenic material used for bone formation applications [2]. rhBMP-2 recruits osteoprogenitor cells to the defected bone area and induces their differentiation into osteoblastic cells [4,5]. Due to its short half-life and rapid loss through diffusion following in vivo administration, a large rhBMP-2 dose is generally required for successful bone formation [5]. However, increasing doses of rhBMP-2 do not lead to proportional increases in bone regeneration. Adipose tissue formation is observed when a large dose of rhBMP-2 is used, leading to a decrease in bone density and quality [4,6]. Immune reactions and paradoxical osteolysis can occur in lumbar posterolateral fusion in clinical applications with high doses of rhBMP-2 [7]. Furthermore, problems have been reported when rhBMP-2 is used at high dosages [8]. Catastrophic ectopic ossification can result from the release of large amounts of rhBMP-2 [7,9]. Therefore, an approach that increases the efficacy of rhBMP-2 in promoting high bone formation without increasing its dosage is needed.

Angiogenesis is necessary for new bone formation because blood vessels can deliver nutrients, oxygen, circulating osteogenic growth and differentiation factors, and osteogenic progenitor cells to new bone formation area [10]. Angiogenic factors such as vascular endothelial growth factor (VEGF), fibroblast growth factor, and platelet-derived growth factor contribute to angiogenesis through endothelial cell proliferation and migration [11]. Furthermore, VEGF directly promotes bone formation by modulating recruitment, survival, and activity of osteoblasts [12]. Therefore, angiogenesis and angiogenic factors are important for bone regeneration. Administration of osteoinductive materials coupled with angiogenic growth factors, especially rhBMP-2 coupled with VEGF, results in enhanced angiogenesis and bone regeneration in critical-size defects in animals [13,14,15,16].

Exosomes are extracellular vesicles ranging in size from to 40-200 nm and are secreted from most cell types [17]. Released exosomes are internalized in target cells and subsequently affect their biological behaviors and promote the tissue regenerative process [18,19,20]. Exosomes secreted from mesenchymal stem cells (MSCs) can promote the proliferation, migration, and tube formation of endothelial cells in vitro, angiogenesis in vivo [21,22,23,24], and in vivo bone regeneration [25,26,27]. The microRNAs (miRNAs) in exosomes play the role of promoting endothelial cell proliferation and vessel formation, and regulating the osteogenic differentiation of targeted MSCs into mature osteoblasts and the subsequent mineralization process [18,28]. In particular, exosomes secreted from umbilical cord MSCs (UCMSCs) [22,23] or human umbilical cord blood [29,30] have shown stimulatory effects on angiogenesis or osteogenesis. Thus, we expected that UCMSCs would be an effective origin for exosomes and exosomes secreted from UCMSCs would be effective to use in angiogenesis and osteogenesis.

Although the therapeutic effects of exosomes are evident, clinical scale production may not be feasible given that cells secrete exosomes at a slow rate [17]. To overcome these limitations, cell-derived nanovesicles (NVs) have been produced by extruding cells through polycarbonate nano-porous membranes. NVs exhibit characteristics (i.e., size and composition) similar to those of exosomes and can be produced in larger quantities at a faster rate [17]. Like exosomes, NVs can also deliver the intercellular contents of donor cells to recipient cells [17]. MSC-derived NVs contain angiogenic proteins and mRNAs and can stimulate in vivo angiogenesis [31].

At higher doses, rhBMP-2 does not necessarily increase bone formation but rather increases the incidence of adverse side effects. Here, we examined whether UCMSC-derived NVs further increase in vivo bone formation at high dose of rhBMP-2. We demonstrated that UCMSC-derived NVs promote the in vitro proliferation, migration, and tube formation of human umbilical vein endothelial cells (HUVECs). They also promote the in vitro migration and osteogenesis of human bone marrow-derived MSCs (hBMSCs). Furthermore, we demonstrated that rhBMP-2 in combination with UCMSC-derived NVs increased in vivo bone formation efficacy compared with that of rhBMP-2 alone in a mouse calvaria defect model. However, UCMSC-derived NVs alone did not induce the in vivo bone formation. Taken together, these data indicated that UCMSC-derived NVs can further increase in vivo bone formation at high dose of rhBMP-2 without posing the risk of adverse effects caused by increasing the rhBMP-2 dose.

## 2. Results

### 2.1. Preparation and Characterization of Ucmsc-Derived Nvs

**Figure 1:**
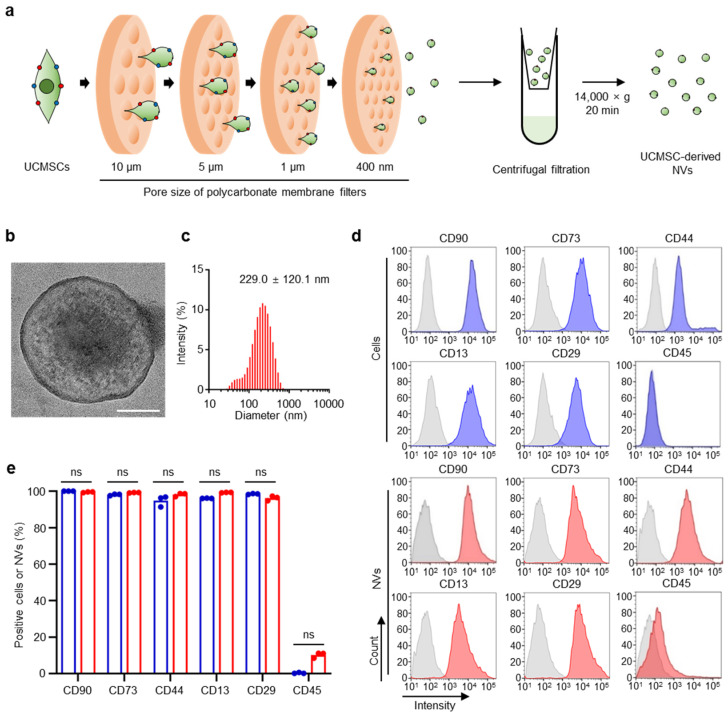
Characterization of umbilical cord mesenchymal stem cell (UCMSC)-derived nanovesicles (NVs). (**a**) Schematic illustration of UCMSC-derived NV fabrication from UCMSCs. (**b**) TEM image of UCMSC-derived NVs. Scale bar, 50 nm. (**c**) Size distribution of UCMSC-derived NVs. (**d**) Representative plots and (**e**) quantification of UCMSC surface marker expression for UCMSCs and UCMSC-derived NVs analyzed by flow cytometry. Blue: cells, red: NVs. Data are presented as means; *n* = 3 per group. The Mann–Whitney test was used for statistical analysis. ns for not significant.

We prepared UCMSC-derived NVs by serial extrusion of UCMSCs. The process of preparing UCMSC-derived NVs is described in Figure 1a. The morphology and size distribution of UCMSC-derived NVs were revealed by transmission electron microscopy (TEM) and dynamic light scattering (DLS) (Figure 1b,c). TEM images demonstrated that the UCMSC-derived NVs had spherical shapes. DLS analysis showed that the sizes (229.0 ± 120.1 nm) were similar to the typical size of extracellular vesicles [32]. Flow cytometric analysis was conducted to identify the expression of MSC cell surface markers in UCMSC-derived NVs compared with those of UCMSCs (Figure 1d). UCMSCs and UCMSC-derived NVs were both positive for MSC cell surface markers (CD73 and CD13) and cell adhesion molecule markers (CD90, CD44, and CD29). Both were negative for hematopoietic markers (CD45; Figure 1e).

### 2.2. Effects of UCMSC-Derived NVs on hBMSCs In Vitro

**Figure 2:**
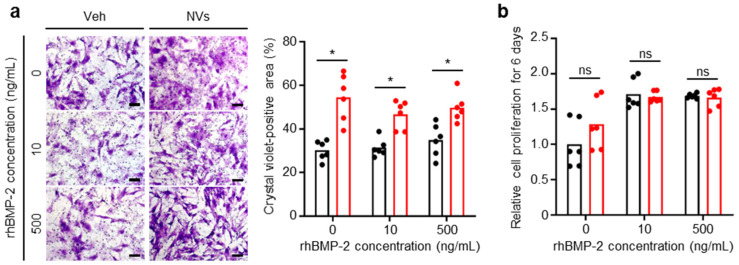
UCMSC-derived NVs promote migration but not proliferation of human bone marrow-derived MSCs (hBMSCs) in vitro. (**a**) Migration and (**b**) proliferation of hBMSCs in the presence or absence of UCMSC-derived NVs at different concentrations of recombinant human bone morphogenetic protein 2 (rhBMP-2). (**a**) Scale bars, 100 µm. Veh indicates vehicle treatment. (**a**,**b**) Data are presented as means; n = 6 per group. Black: Veh, Red: NVs. The Mann–Whitney test was used for statistical analysis. * *p* < 0.05. ns for not significant.

hBMSCs were cultured in the presence of different concentrations (0, 10, and 500 ng/mL) of rhBMP-2 with or without UCMSC-derived NVs. The choice of the rhBMP-2 concentrations was based on a previous study that revealed a rhBMP-2 concentration of 500 ng/mL exhibited the highest effect on in vitro osteogenic differentiation in rat bone marrow cells [33]. UCMSC-derived NVs were effective for the migration of hBMSCs. The effect of UCMSC-derived NVs on the migration of hBMSCs was analyzed by the Transwell Chamber assay. Figure 2a shows a larger crystal violet-positive area, which indicates migrated hBMSCs, in the UCMSC-derived NVs-treated groups regardless of the rhBMP-2 concentration. To examine the effects of UCMSC-derived NVs on the proliferation of hBMSCs, a cell counting kit-8 (CCK-8) assay was performed after six days of treatment. UCMSC-derived NVs did not affect the proliferation of hBMSCs at various concentrations of rhBMP-2 (Figure 2b).

### 2.3. Effects of UCMSC-Derived NVs on Osteogenesis of hBMSCs and MC3T3-E1 Cells In Vitro

**Figure 3:**
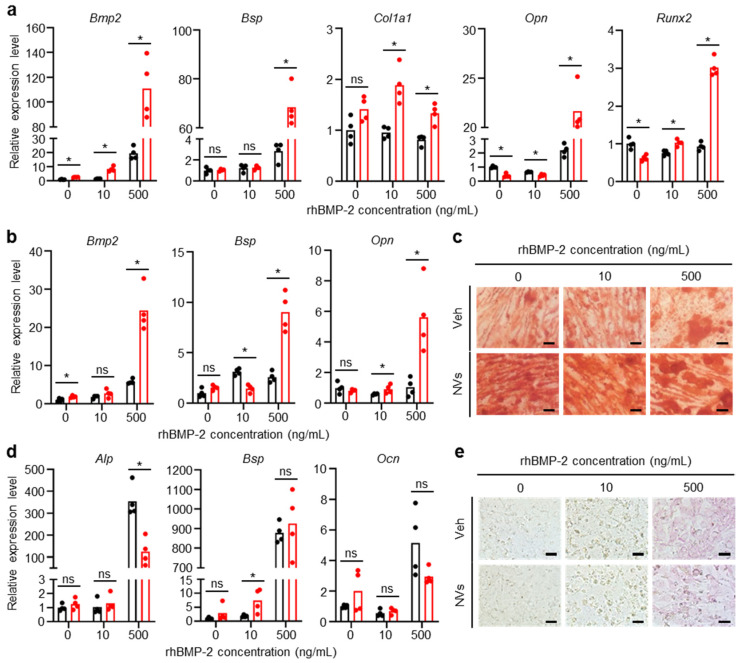
UCMSC-derived NVs promote osteogenesis of hBMSCs, but not MC3T3-E1 cells in the presence or absence of rhBMP-2 in vitro. (**a**) mRNA levels 11 days and (**b**) 21 days after, and (**c**) Alizarin red S staining 18 days after inducing osteogenesis of hBMSCs in the presence or absence of UCMSC-derived NVs at different concentrations of rhBMP-2. (**d**) mRNA levels 7 days after and (**e**) alkaline phosphatase (ALP) staining 10 days after inducing osteogenesis of MC3T3-E1 cells in the presence or absence of UCMSC-derived NVs at different concentrations of rhBMP-2. (**a**,**b**,**d**) Black: Veh, Red: NVs. Data are presented as means; *n* = 4 per group. The Mann–Whitney test was used for statistical analysis. * *p* < 0.05. ns for not significant. (**c**,**e**) Scale bars, 50 µm. Veh indicates vehicle treatment.

To evaluate the effect of UCMSC-derived NVs on osteogenesis, hBMSCs, and MC3T3-E1 cells (an osteoblastic cell line) were used. Primer sequences used for quantitative real-time polymerase chain reaction (qRT-PCR) are shown in Table S1. The mRNA expression levels of osteogenic markers, Bmp2, bone sialoprotein (Bsp), collagen type I alpha 1(Col1a1), osteopontin (Opn), and runt-related transcription factor 2 (Runx2), in hBMSCs were analyzed at 11 days and 21 days after osteogenic induction in the presence of different concentrations (0, 10, and 500 ng/mL) of rhBMP-2 with or without UCMSC-derived NVs (Figure 3a,b). Compared with the vehicle(Veh)-treated groups, the UCMSC-derived NV-treated groups showed higher expression of the osteogenic markers at a high rhBMP-2 concentration (500 ng/mL). Alizarin red S staining was performed after 18 days of induction to evaluate the osteogenesis in hBMSCs qualitatively (Figure 3c). There were stark differences between the Veh and UCMSC-derived NVs-treated groups. The results confirmed that UCMSC-derived NVs promoted osteogenesis of hBMSCs. To further evaluate the effects of NVs on osteogenesis, Veh and UCMSC-derived NVs were treated with hBMSCs in the absence of rhBMP-2 in non-osteogenesis induction medium. After two days of induction, mRNA levels of alkaline phosphatase (Alp), Bsp, osteocalcin (Ocn), Opn, and Runx2 showed no difference between the Veh-treated and the UCMSC-derived NVs-treated groups (Figure S1a). Moreover, the mRNA levels of the UCMSC-derived NVs-treated group decreased more after seven days and were significantly different from the Veh-treated group (Figure S1b). Alkaline phosphatase (ALP) staining and Alizarin red S staining after 14 days of osteogenesis induction confirmed that UCMSC-derived NVs were not effective in the osteogenesis of hBMSCs in a non-osteogenic environment (Figure S1c).

The mRNA expression levels of osteogenic markers, Alp, Bsp, and Ocn, in MC3T3-E1 cells were analyzed seven days after osteogenic induction in the presence of different concentrations (0, 10, and 500 ng/mL) of rhBMP-2 with or without UCMSC-derived NVs (Figure 3d). The mRNA levels differed only for Alp between the Veh and UCMSC-derived NVs-treated groups. Furthermore, ALP staining was performed after 10 days of treatment to evaluate the osteogenesis qualitatively (Figure 3e). The results indicated that UCMSC-derived NVs were not effective in the osteogenesis of MC3T3-E1 cells.

### 2.4. Effects of UCMSC-Derived NVs on HUVECs In Vitro

**Figure 4:**
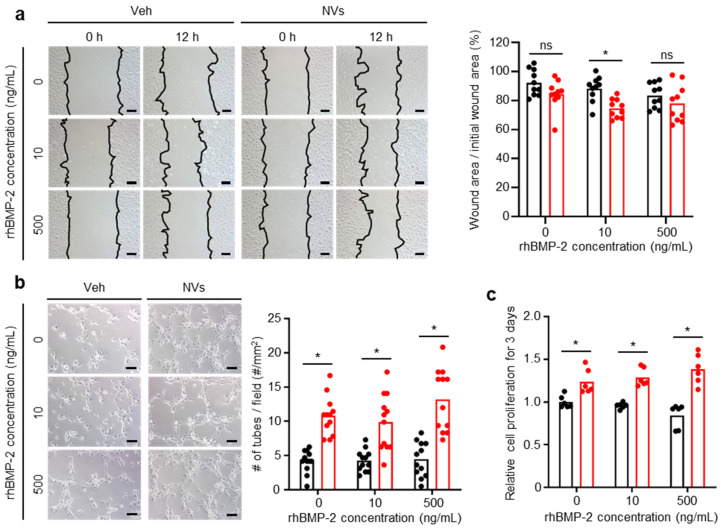
UCMSC-derived NVs promote tube formation and proliferation of human umbilical vein endothelial cells (HUVECs) in the presence or absence of rhBMP-2 in vitro. (**a**) Migration, (**b**) tube formation, and (**c**) proliferation of HUVECs in the presence or absence of UCMSC-derived NVs at different concentrations of rhBMP-2. (**a**) Scale bars, 500 µm. *n* = 10 per group. (**b**) Scale bars, 100 µm. *n* = 12 per group. (**c**) *n* = 6 per group. (**a**,**b**) Veh indicates vehicle treatment. (**a**–**c**) Black: Veh, Red: NVs. Data are presented as means. The Mann–Whitney test was used for statistical analysis. * *p* < 0.05. ns for not significant.

The HUVECs were cultured identical to the hBMSCs. To determine the effects of UCMSC-derived NVs on the migration of HUVECs, scratch wound healing assays were performed. Images of the initial wound area and wound area 12 h after the treatment are shown in Figure 4a. The ratio of the wound area relative to the initial wound area showed that in the presence of UCMSC-derived NVs at 10 ng/mL of rhBMP-2, HUVECs migrated faster significantly, indicating that UCMSC-derived NVs promoted the migration of HUVECs. Tube formation assays were performed in Matrigel-coated wells to examine the pro-angiogenic effect of UCMSC-derived NVs (Figure 4b). After 4 h of incubation in the presence of UCMSC-derived NVs, HUVECs formed significantly more tube structures than the Veh groups. The CCK-8 assay was performed after three days of treatment to examine the effects of UCMSC-derived NVs on the proliferation of HUVECs (Figure 4c). The results showed that UCMSC-derived NVs enhanced the proliferation of HUVECs. Taken together, UCMSC-derived NVs were effective in the migration, tube formation, and proliferation of HUVECs.

### 2.5. Effects of UCMSC-Derived NVs on In Vivo Bone Formation

**Figure 5:**
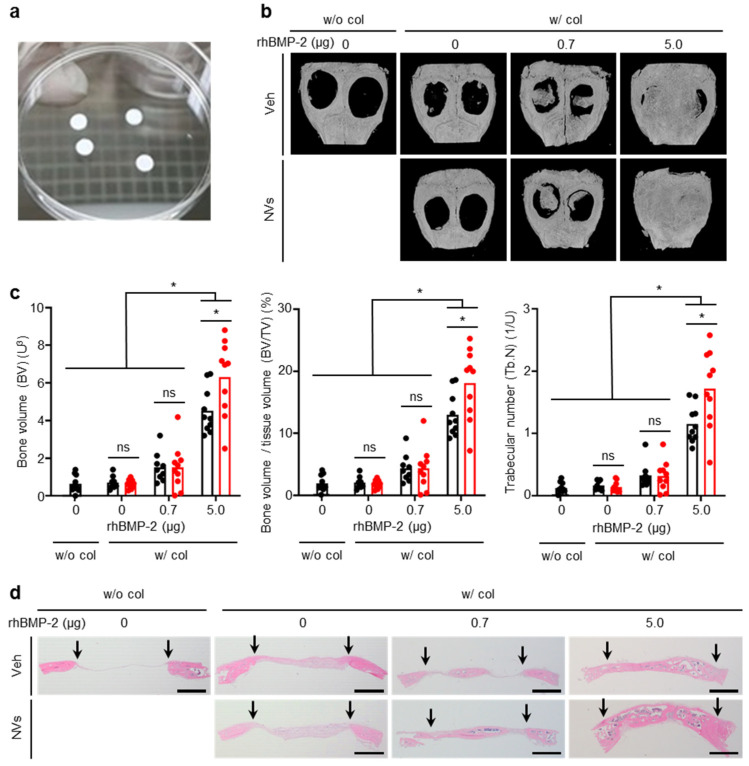
UCMSC-derived NVs promote bone formation in calvaria defects of mice at 6 weeks. (**a**) Image of collagen sponge with 4 mm diameter used for implantation. (**b**) The calvaria scanned by micro-CT. Veh indicates vehicle treatment. (**c**) Bone volume (BV), bone volume to tissue volume ratio (BV/TV) and trabecular number (Tb.N) evaluated with micro-CT. Black: Veh, Red: NVs. Data are presented as means; *n* = 8 for w/col-Veh-0. *n* = 9 for w/col-Veh-0.7. *n* = 10 for the other groups. One-way ANOVA with Tukey’s post-test was used for statistical analysis. * *p* < 0.05. ns for not significant. (**d**) Cross-sectional views of the calvaria defects stained with hematoxylin and eosin (H&E). Arrows indicate defect margin. Scale bars, 1 mm. (**b**–**d**) w/o col: without collagen sponge, w/col: with collagen sponge.

To evaluate the effects of UCMSC-derived NVs on in vivo bone formation, different doses (0, 0.7, and 5.0 µg) of rhBMP-2 with or without UCMSC-derived NVs were soaked in collagen sponges 4 mm in diameter and 12 µL in volume (Figure 5a) that served as scaffolds for bone formation. The rhBMP-2 dose of 5.0 µg used for a mouse bone defect model (4 mm-diameter) was considered as the high dose, as the maximal rhBMP-2 concentration necessary for inducing effective bone formation in rodents is 0.4 mg/mL [8]. The rhBMP-2 dose of 0.7 µg was chosen as the lower dose since this dose was expected to fill 50% of the calvaria defect area in mice [5]. Micro-computed tomography (micro-CT) images showed bone formation in the calvaria defects eight weeks after the implantation of collagen sponges (Figure 5b). No infections or tissue necrosis were observed. In the w/o (without) col (collagen sponge) group, w/(with) col-0-Veh group, and w/col-0-NVs group, the bone was not formed in the calvaria defects. Small mineralized bones were generated in the w/col-0.7-Veh and w/col-0.7-NVs group, but these bone masses were insufficient to cover the defects. Moreover, there was no significant difference in the volume of new bone between the two groups. In contrast, the newly formed bone covered the defect partially or completely in the two groups at 5.0 µg of rhBMP-2. Importantly, the amount of newly formed bone induced with UCMSC-derived NVs was much higher than that treated with rhBMP-2 alone. The difference between the two groups was statistically significant in bone volume (BV), bone volume/tissue volume (BV/TV), and trabecular number (Tb.N; Figure 5c). Hematoxylin and eosin (H&E) staining of histological analyses revealed the same trend as the results of the micro-CT analysis for bone formation (Figure 5d). In the w/o col group, w/col-0-Veh group, and w/col-0-NVs group, fibrous tissue was generated in the defects and no bone was formed. At 5.0 µg rhBMP-2, the UCMSC-derived NVs group resulted in much more bone formation compared with the Veh group. Small new bone masses were observed in the two groups at 0.7 µg rhBMP-2. No residual scaffold was observed in the defects at six weeks.

### 2.6. Effects of UCMSC-Derived NVs on In Vivo Angiogenesis

**Figure 6:**
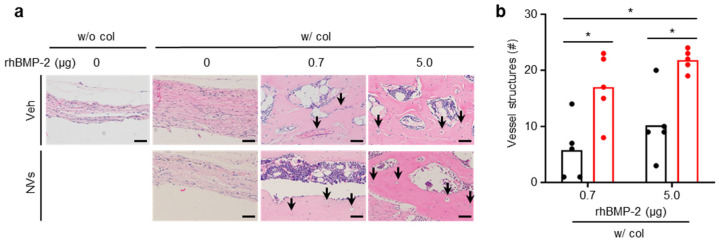
UCMSC-derived NVs promote angiogenesis in calvaria defects of mice at 6 weeks. (**a**) Cross-sectional views of the calvaria defects stained with H&E. Veh indicates vehicle treatment. Arrows indicate vessel structures. Scale bars, 50 µm. (**b**) Quantification of vessel structures in the defects. Black: Veh, Red: NVs. Data are presented as means; *n* = 5 per group. One-way ANOVA with Tukey’s post-test was used for statistical analysis. * *p* < 0.05. (**a**,**b**) w/o col: without collagen sponge, w/col: with collagen sponge.

Vessel structures in the newly formed bone areas are shown in Figure 6a. The number of vessel structures was quantified by histological analysis (Figure 6b). The standards for determination of vessel structures were flat morphology of endothelial cells, tube structure, and structure filled with red blood cells [34]. The vessel structures were counted as positive when the structure satisfied all these standards. Since bone regeneration was not observed in the groups with no rhBMP-2 treatment, the quantification was performed only in the rhBMP-2-treated groups. The number of vessel structures was higher in the UCMSC-derived NVs-treated groups, regardless of the rhBMP-2 dose. This indicates that the formation of vessel structures was affected by UCMSC-derived NVs.

## 3. Discussion

Both osteoblastic differentiation of MSCs and angiogenesis promote in vivo bone formation [10,28]. The stimulatory effects of exosomes secreted from MSCs on angiogenesis and osteogenesis have been demonstrated in previous studies [28,35]. Exosomal miRNAs such as miR-129, miR-136, and the miR-17-92 cluster promote endothelial cell proliferation and vessel formation [28]. Exosomal miRNAs such as miR-199b, miR-218, miR-148a, miR-135b, miR-203, miR-219, miR-299-5p, and miR-302b are upregulated during the osteoblastic differentiation of MSCs [28]. Exosomal miRNAs such as miR-221, miR-155, miR-885-5p, miR-181a, and miR-320c are downregulated during the osteoblastic differentiation of MSCs [28]. Furthermore, UCMSCs are known to express pro-angiogenic factors, such as VEGF [35]. In this study, we used UCMSC-derived NVs, which were produced by extrusion of UCMSCs, rather than exosomes secreted from UCMSCs. It is known that total RNA profiles of origin cells, exosomes secreted from the cells, and the cell-derived NVs are similar [17]. Thus, VEGF and similar miRNAs in UCMSC-derived NVs are likely responsible for the enhanced bone formation in this study.

Although the exact components in UCMSC-derived NVs that support in vivo bone formation are have not yet been identified, this study revealed that UCMSC-derived NVs promote migration and osteogenic differentiation of hBMSCs and migration and angiogenesis of HUVECs in vitro. UCMSC-derived NVs showed a synergy effect on rhBMP-2-mediated in vivo bone formation. UCMSC-derived NVs can promote migration of MSCs from neighboring tissue to bone defect regions. Migrated MSCs can be induced to differentiate into osteogenic cells at the region of bone formation by rhBMP-2 and UCMSC-derived NVs. VEGF and other pro-angiogenic factors in UCMSC-derived NVs can support the attraction of endothelial cells to the bone defect region and subsequent blood vessel formation [36,37]. After the initiation of bone formation, a cascade of vascular invasion and ossification leads to the bone expansion [36]. Meanwhile, in a non-osteoinductive environment, UCMSC-derived NVs did not promote the in vitro osteogenesis of MSCs. Similarly, UCMSC-derived NVs did not promote the in vivo bone formation when rhBMP-2 was not used. These results suggested that UCMSC-derived NVs potentiate the osteogenic efficacy of rhBMP-2.

In the body, endothelial cells, osteoblasts, and MSCs regenerate bone by interacting with each other. Angiogenic factors such as VEGF influence osteoblasts indirectly by affecting endothelial cells in the bone regeneration process [38]. rhBMP-2 enhances angiogenesis by activating endothelial cells and inducing osteoblast-derived VEGF expression [15]. Therefore, identifying the role of UCMSC-derived NVs and rhBMP-2 in the interactions between endothelial cells and osteoblasts or MSCs would lead to establishment of their role in bone formation.

Regarding dose of UCMSC-derived NVs for the in vivo bone formation study, a previous study used extracellular vesicles secreted from MSCs, loaded in a scaffold at a concentration of 2.22 µg per 1 µL scaffold for in vivo bone formation [24]. We used the same concentration (30 µg of UCMSC-derived NVs per 13.5 µL scaffold) for the in vivo bone formation, which was a maximum of 5.0 µg rhBMP-2 and 30 µg NVs. We used the same ratio of rhBMP-2 to UCMSC-derived NVs (5:30) for the in vitro osteogenic differentiation study (maximum 0.5 µg/mL rhBMP-2 and 3 µg/mL UCMSC-derived NVs). For assays using HUVECs, we used two different concentrations of UCMSC-derived NVs, 3 µg/mL and 20 µg/mL, to examine the effect of NV concentration. The data showed that UCMSC-derived NVs at 3 µg/mL and 20 µg/mL showed the same tendency. We chose to display the results of 20 µg/mL UCMSC-derived NVs in assays using HUVECs.

Potential limitations of NVs should be considered for the clinical application of NVs. Exosomes have in vivo limitations of their short half-life and rapid clearance by the immune system [35]. As NVs are characteristically similar to exosomes, the stability and retention ability of NVs in vivo is important for clinical applications. The development of appropriate administration methods should be considered to facilitate effective uptake of NVs [35].

Administering large doses of rhBMP-2 is considered risky in clinical applications. To achieve higher bone formation without using a high dosage of rhBMP-2, we introduced the use of UCMSC-derived NVs. Unlike previous studies that have used exosomes secreted from multiple other types of MSCs, this study simultaneously revealed that UCMSCs can be used as a material for enhancing osteogenesis, and exosomes can be successfully alternated to NVs extruded from cells. Moreover, we expected that UCMSC-derived NVs may present therapeutic promise in other diseases that require effective angiogenic function.

## 4. Materials and Methods

### 4.1. Cell Culture

UCMSC (CEFO, Seoul, Korea), hBMSC (Lonza, Basel, Switzerland), MC3T3-E1 (ATCC, Manassas, VA, USA), and HUVEC (Lonza) were purchased. UCMSCs were cultured in Dulbecco’s modified Eagle’s medium (DMEM) with low glucose (Gibco, Carlsbad, CA, USA), hBMSCs in DMEM with high glucose (Gibco), MC3T3-E1 cells in minimum essential medium alpha (MEM alpha; Gibco) supplemented with 10% fetal bovine serum (FBS; Gibco), 100 U/mL penicillin and 100 µg/mL streptomycin (P/S; Gibco). The HUVECs in endothelial cell growth medium-2 (EGM-2) with a supplement (Lonza) were cultured at 37 °C with 5% CO_2_. Cell passaging was performed when a monolayer of adherent cells reached 80% confluence.

### 4.2. Preparation of UCMSC-Derived NVs

To prepare UCMSC-derived NVs, UCMSCs from the seventh passage were detached by scraping after removing the medium. The cell suspension was sequentially extruded five times each through 10, 5, 1 µm, and 400 nm polycarbonate membrane filters (Whatman, Maidstone, UK) using a mini extruder (Avanti, Alabaster, AL, USA). To collect NVs, the suspension of NVs was centrifuged at 14,000× *g* for 20 min using Amicon 3k Ultra Centrifugal filters (Millipore, Burlington, MA, USA). NVs were filtered through a 0.22 µm filter and stored at −80 °C until use. The concentration of NVs was determined by the Bradford assay with Bradford reagent (Sigma, St. Louis, MO, USA). The morphology of the UCMSC-derived NVs was evaluated using TEM (JEM-F200, JEOL Ltd., Tokyo, Japan).

### 4.3. UCMSC-Derived NVs Size

The size and size distribution of UCMSC-derived NVs were measured by DLS (Zeta-sizer Nano ZS, Malvern Instruments, Malvern, UK). UCMSC-derived NVs were dispersed in distilled water and the particle size and size distribution were measured and analyzed using Zetasizer software (version 7.13, Malvern Instruments).

### 4.4. Flow Cytometry Analysis

The adherent UCMSCs and UCMSC-derived NVs were collected, washed with and suspended in a cell staining buffer (Biolegend, San Diego, CA, USA). Both cells and NVs were stained with anti-human CD90 FITC (03011-50-25, Biogems, Westlake Village, CA, USA, 1:20), anti-human/mouse CD44 APC (06511-80-25, Biogems, 1:160), anti-human CD45 FITC (07151-50-25, Biogems, 1:20), anti-human CD73 PE (05811-60-25, Biogems, 1:20), anti-human CD13 APC (05211-80-25, Biogems, 1:20), and anti-human CD29 FITC (11311-50-25, Biogems, 1:20) according to the manufacturer’s instructions. UCMSC-derived NVs were washed with cell staining buffer (Biolegend) and collected by centrifugation at 27,237× *g* for 30 min. 10^4^ cells or 5 × 10^3^ NVs were used for each repeated analysis. FACS AriaII (BD Biosciences, San Jose, CA, USA) and FlowJo software (version 10, BD Biosciences) were used to analyze the data.

### 4.5. Cell Proliferation Assay

HUVECs from the eighth passage were seeded at a density of 3 × 10^3^ cells/cm^2^ in a 24-well plate and cultured in EGM-2 without supplementation in the presence or absence of NVs (20 µg/mL) with different concentrations of rhBMP-2 (10 or 500 ng/mL, CGbio, Seongnam, Korea). At three days post-treatment [24], the cell culture medium was removed and the CCK-8 (DoGen, Seoul, Korea) was used according to the manufacturer’s instructions. Briefly, CCK-8 solution with fresh cell culture medium was added and maintained for another 2 h. The optical density at 450 nm was measured using a Powerwave X340 microplate reader (BIO-TEK Instruments, Winooski, VT, USA). hBMSCs from the seventh passage were seeded at a density of 1.875 × 10^3^ cells/cm^2^ in a 24-well plate and cultured in DMEM with high glucose supplemented with 10% FBS and P/S in the presence or absence of NVs (3 µg/mL) with different concentrations of rhBMP-2 (10 or 500 ng/mL). At six days post-treatment [24], CCK-8 assays were performed as described above.

### 4.6. hBMSCs Migration Assay

hBMSCs from the eighth passage in DMEM with high glucose without supplementation were dispersed within the upper chamber of 24-well Transwell dishes with 8 µm polycarbonate membranes (Corning Inc., Corning, NY, USA) at a density of 7.5 × 10^4^ cells/cm^2^. DMEM with high glucose without supplementation in the presence or absence of NVs (3 µg/mL) with different concentrations of rhBMP-2 (10 or 500 ng/mL) were placed in the lower chamber. After 24 h of incubation, the surface of the membrane was rinsed with phosphate-buffered saline (PBS) and wiped with a cotton bud. The membrane was stained with 0.1% crystal violet (Sigma) and the crystal violet-positive area was measured with Image J software (version 1.51k, National Institutes of Health, Bethesda, MD, USA) [39].

### 4.7. Osteogenic Differentiation of MC3T3-E1 Cells and hBMSCs

For osteogenic differentiation, hBMSCs from the fifth passage were seeded at a density of 1.5 × 10^3^ cells/cm^2^ in a 6-well plate and cultured in DMEM with high glucose supplemented with 10% FBS and P/S until reaching 80% confluence. hBMSCs were then cultured in DMEM with high glucose supplemented with 10% FBS, P/S, 10 mM β-glycerophosphate (Sigma), 100 nM dexamethasone (Sigma-Aldrich, St. Louis, MO, USA), and 50 mg/L ascorbic acid (Sigma) in the presence or absence of NVs (3 µg/mL) with different concentrations of rhBMP-2 (10 or 500 ng/mL) [40]. The medium was changed twice a week for 21 days. Cells were evaluated to identify the level of osteogenesis on days 11 and 21. MC3T3-E1 cells from the 13th passage were seeded at a density of 3 × 10^3^ cells/cm^2^ in a 6-well plate and cultured in MEM Alpha supplemented with 10% FBS and P/S until reaching 80% confluence. MC3T3-E1 cells were then cultured in MEM Alpha supplemented with 5% FBS, P/S, 3 mM β-glycerophosphate, and 50 mg/L ascorbic acid in the presence or absence of NVs (3 µg/mL) with different concentrations of rhBMP-2 (10 or 500 ng/mL) [41,42]. The medium was changed twice a week for 13 days. Cells were evaluated to identify the level of osteogenesis on days seven and 13.

### 4.8. ALP and Alizarin Red S Staining

Osteogenesis-induced MC3T3-E1 cells and hBMSCs were washed twice in PBS and fixed in 4% paraformaldehyde (PFA) for 15 min. MC3T3-E1 cells were then stained with an ALP detection kit (Sigma-Aldrich) according to the manufacturer’s instructions. Briefly, cells were incubated with ALP staining solution for 15 min in the dark. hBMSCs were stained with 2% Alizarin red S (Sigma-Aldrich) solution (pH 4.1–4.3) for 20 min [43]. After staining, cells were washed with PBS three times and imaged under a light microscope (IX71, Olympus, Tokyo, Japan).

### 4.9. qRT-PCR

To identify the level of osteogenesis in MC3T3-E1 cells and hBMSCs, total RNA from osteogenesis-induced cells was extracted using QIAzol (Qiagen, Hilden, Germany). Extracted RNA was reverse transcribed to cDNA using RT premix (Bioneer, Daejeon, Korea) according to the manufacturer’s protocol. qRT-PCR was performed using the StepOnePlus Real-Time PCR System (Applied Biosystems, Foster City, CA, USA) with TOPreal™ qPCR 2X PreMIX (Enzynomics, Daejeon, Korea) according to the manufacturer’s protocol. Relative gene expression was calculated by the 2-∆∆Ct method, in which the expression levels of target genes were normalized to glyceraldehyde 3-phosphate dehydrogenase. Primers used for qRT-PCR are presented in the Table S1.

### 4.10. Scratch Wound Healing Assay

HUVECs from the seventh passage were seeded at a density of 10^4^ cells/cm^2^ in a 6-well plate and cultured in EGM-2 with supplement until the monolayer of adherent cells reached 100% confluence. A wound was created in the middle of the plate by manually scraping the cell monolayer with a P1000 pipette tip. The cells were then cultured in EGM-2 without supplementation in the presence or absence of NVs (20 µg/mL) with different concentrations of rhBMP-2 (10 or 500 ng/mL; CGbio, Seongnam, Korea) for 12 h [24]. The wounded region at 0 and 12 h after the scratch was captured using a microscope (IX71, Olympus, Tokyo, Japan). The area of the wounded region was measured using Image J software (version 1.51k, National Institutes of Health).

### 4.11. Tube Formation Assay

HUVECs from the eighth passage were seeded into Matrigel (Corning)-coated 12-well plates at a density of 3.75 × 10^4^ cells/cm^2^ and cultured in EGM-2 without supplementation in the presence or absence of NVs (20 µg/mL) with different concentrations of rhBMP-2 (10 or 500 ng/mL) for 4 h [24]. Tube formation was captured as images by microscope (IX71, Olympus). The number of closed tube structures in the standard area was counted.

### 4.12. Mouse Calvaria Defect Model and Implantation

Collatape (Zimmer, Warsaw, IN, USA) was used as a scaffold to load UCMSC-derived NVs and rhBMP-2. Thirty-five six-week-old male athymic nude mice (Koatech, Pyeongtaek, Korea) were divided into seven groups (five mice in each group, two calvaria defects per mouse) as follows: without collatape group (w/o col), collatape only group (w/col-0-Veh), NVs group (w/col-0-NVs), 0.7 µg rhBMP-2 group (w/col-0.7-Veh), 0.7 µg rhBMP-2 with NVs group (w/col-0.7-NVs), 5.0 µg rhBMP-2 group (w/col-5.0-Veh), and 5.0 µg rhBMP-2 with NVs group (w/col-5.0-NVs). For NVs-treated groups, 30 µg of UCMSC-derived NVs were treated. Mice were anesthetized with Zoletil^®^ (50 mg/kg, Virbac, Korea) and Rompun^®^ (25 mg/kg, Bayer, Korea). After sterilization with betadine, a longitudinal incision was made on the midline of the frontal and parietal bone, and the periosteum was removed to expose the parietal bone surface. Two 4 mm-diameter circular transosseous defects were made using a surgical trephine bur in the parietal bone and rinsed with PBS to eliminate the bone debris. Then, the calvaria defects were filled with or without scaffolds according to the group arrangement. After implantation, skin incisions were sutured and sterilized. The 4 mm-diameter bone defect was considered a critical size for the mouse calvarial bone defect model [44]. This research was approved by the Institutional Animal Care and Use Committee of the Seoul Metropolitan Government Boramae Medical Center of Seoul National University (IACUC No. 2018-0016, 28 May 2018).

### 4.13. Micro-CT

The mice were sacrificed eight weeks after the implantation, and the mice skulls were harvested and fixed in 4% PFA. Micro-CT (Skyscan 1172, Bruker, Kontich, Belgium) was performed on the calvaria sample to evaluate bone formation at a voltage of 59 kV, a current of 167 µA, and a resolution of 9.90 µm/pixel with a 0.5 mm aluminum filter. The raw micro-CT images were reconstructed using NRecon (version 1.7.0.4, Bruker) with ring artifact and beam hardening corrections. The 4 mm-diameter calvaria defect was selected as the region of interest (ROI). The parameters of new bone in the volume of interest (VOI; 280 slices of ROI) were obtained using the CTAn program (version 1.17.7.2, Bruker).

### 4.14. H&E Staining

The calvaria specimens were decalcified with 10% ethylenediaminetetraacetic acid (pH 7.4) until the bones were easily penetrated by a needle without any force [45]. The specimens were embedded in paraffin and sectioned at 4-μm thickness in the coronal plane through the defect center using a microtome (Leica Microsystems, Wetzlar, Germany). According to a general protocol, sections were stained with H&E (BBC biochemical, Mount Vernon, WA, USA). The newly formed bones in the calvaria defects were observed using a light microscope (DP74, Olympus), and the vessel-like structures in the new bone were quantified using Image J software (version 1.51k, National Institutes of Health).

### 4.15. Statistical Analysis

Cellular images used for quantification were taken from two or three randomly selected imaging fields for each experimental condition. Image J software was used to quantify the positive signal by intensity. No samples or animals were excluded from the analysis, except animals who died prematurely. For micro-CT data performed in vivo, each data point represents a defect. For data associated with H&E staining, each data point represents a single mouse. Image J software was used to quantify in vivo data. All statistical analyses were performed using the GraphPad Prism software (version 8.02, GraphPad Software, San Diego, CA, USA). All data were expressed as means or means ± SD. Statistical significance was assessed using one-way ANOVA with Tukey’s post-test or the Mann–Whitney test.

## Supplementary Materials

Supplementary materials can be found at https://www.mdpi.com/1422-0067/21/17/6425/s1, Figure S1: UCMSC-derived NVs do not promote osteogenesis of hBMSCs in non-osteogenesis induction condition, Table S1: Primer sequences used for qRT-PCR.

## Abbreviations

rhBMP-2	Recombinant human bone morphogenetic protein 2
VEGF	Vascular endothelial growth factor
MSC	Mesenchymal stem cell
miRNAs	microRNAs
NVs	Nanovesicles
UCMSC	Umbilical cord mesenchymal stem cell
HUVEC	Human umbilical vein endothelial cell
hBMSC	Human bone marrow-derived mesenchymal stem cell
TEM	Transmission electron microscopy
DLS	Dynamic light scattering
ALP	Alkaline phosphatase
qRT-PCR	Quantitative real-time polymerase chain reaction
Bsp	Bone sialoprotein
Col1a1	Collagen type I alpha 1
Ocn	Osteocalcin
Opn	Osteopontin
Runx2	Runt-related transcription factor 2
Micro-CT	Micro-computed tomography
BV	Bone volume
BV/TV	Bone volume/tissue volume
Tb.N	Trabecular number
H&E	Hematoxylin and eosin
DMEM	Dulbecco’s modified Eagle’s medium
MEM	Minimum essential medium
EGM-2	Endothelial cell growth medium-2
FBS	Fetal bovine serum
P/S	Penicillin and streptomycin
CCK-8	Cell counting kit-8
PBS	Phosphate-buffered saline
PFA	Paraformaldehyde
ROI	Region of interest
VOI	Volume of interest
