# Supplementary material for: Umbilical Cord Mesenchymal Stem Cell-Derived Nanovesicles Potentiate the Bone-Formation Efficacy of Bone Morphogenetic Protein 2
Source: Int J Mol Sci. 2020 Sep 3;21(17):6425. doi: 10.3390/ijms21176425 (PMC7504262; doi:10.3390/ijms21176425)
Supplement: Supplementary file 1 [file ijms-21-06425-s001.zip › ijms-901766-supplementary/200731 IJMS_bmp-2_supplementary data.docx]

Supporting Information


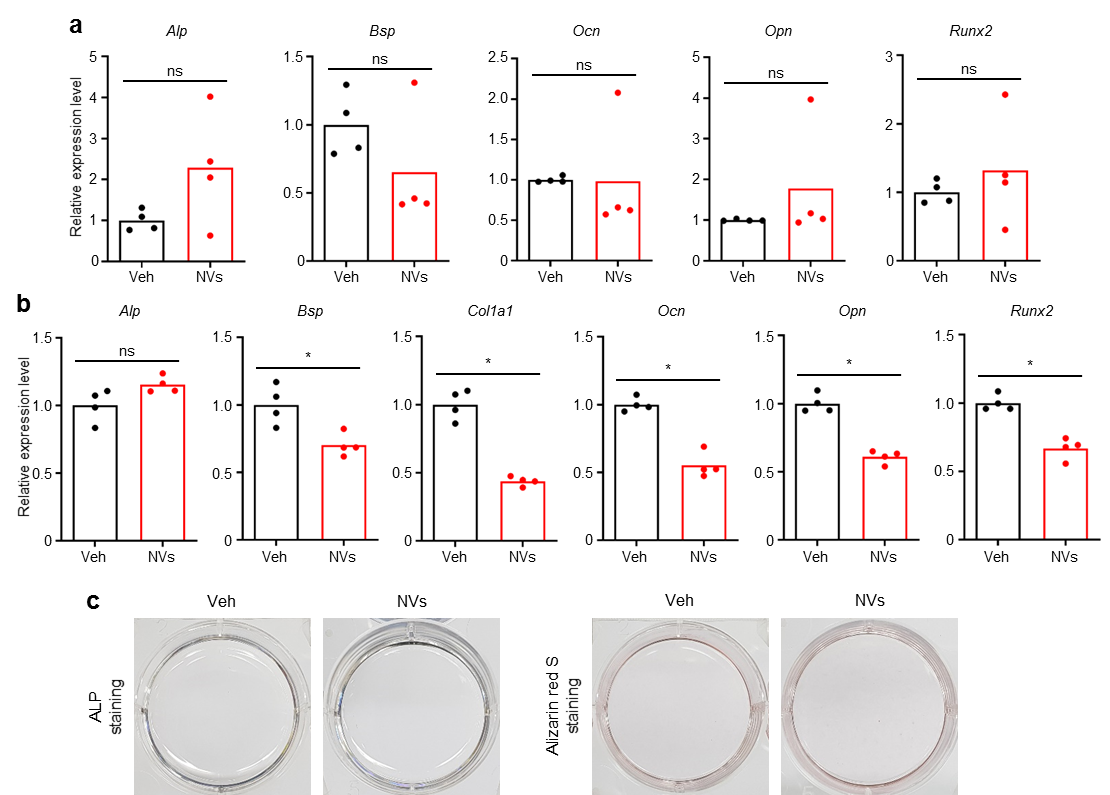


**Figure 1.** UCMSC-derived NVs do not promote osteogenesis of hBMSCs in non-osteogenesis induction condition. (**a**) mRNA levels on day 2 and (**b**) day 7, and (**c**) ALP staining and Alizarin red S staining on day 14 of hBMSCs in the presence or absence of UCMSC-derived NVs without rhBMP-2 in non-osteogenesis induction medium. (**a-b**) Data are the mean. *n* = 4 per group. Mann-Whitney test was used for statistical analysis. *P<0.05. ns for not significant.

**Table 1.** Primer sequences used for qRT-PCR.

| **Gene**  **(mouse)** | **Forward primer (5’-3’)** | **Reverse primer (5’-3’)** |
| --- | --- | --- |
| *Alp* | GCTGATCATTCCCACGTTTT | CTGGGCCTGGTAGTTGTTGT |
| *Bsp* | AAGAGGAAGAAAATGAGAACGA | GCTTCTTCTCCGTTGTCTCC |
| *Col1a1* | AACGAGATCGAGCTCAGAGG | GACTGTCTTGCCCCAAGTTC |
| *Ocn* | CCGGGAGCAGTGTGAGCTTA | AGGCGGTCTTCAAGCCATACT |
| *Opn* | TCAGGACAACAACGGAAAGGG | GGAACTTGCTTGACTATCGATCAC |
| *Runx2* | GCCGGGAATGATGAGAACTA | GGACCGTCCACTGTCACTTT |
| *Gapdh* | AACTTTGGCATTGTGGAAGG | ACACATTGGGGGTAGGAACA |
|  |  |  |
| **Gene**  **(human)** | **Forward Primer (5’-3’)** | **Reverse Primer (5’-3’)** |
| *Alp* | CCTCGTTGACACCTGGAAGAG | TTCCGTGCGGTTCCAGA |
| *Bmp2* | GGAGAAGGAGGAGGCAAG | GACACGTCCATTGAAAGAGC |
| *Bsp* | CAGGCCACGATATTATCTTTACA | CTCCTCTTCTTCCTCCTCCTC |
| *Col1a1* | CAGCCGCTTCACCTACAGC | TTTTGTATTCAATCACTGTCTT |
| *Ocn* | TGTGAGCTCAATCCGGACTGT | CCGATAGGCCTCCTGAAAGC |
| *Opn* | TGAGCATTCCGATGTGATTGA | TGTGGAATTCACGGCTGACTT |
| *Runx2* | CCAGATGGGACTGTGGTTACTG | TTCCGGAGCTCAGCAGAATAA |
| *Gapdh* | GCTGAACGGGAAGCTCACTGGCA | AGGTCAGGTCCACCACTGACACG |
