# Supplementary figures and images for: Umbilical Cord Mesenchymal Stem Cell-Derived Nanovesicles Potentiate the Bone-Formation Efficacy of Bone Morphogenetic Protein 2
Source: Int J Mol Sci. 2020 Sep 3;21(17):6425. doi: 10.3390/ijms21176425 (PMC7504262; doi:10.3390/ijms21176425)

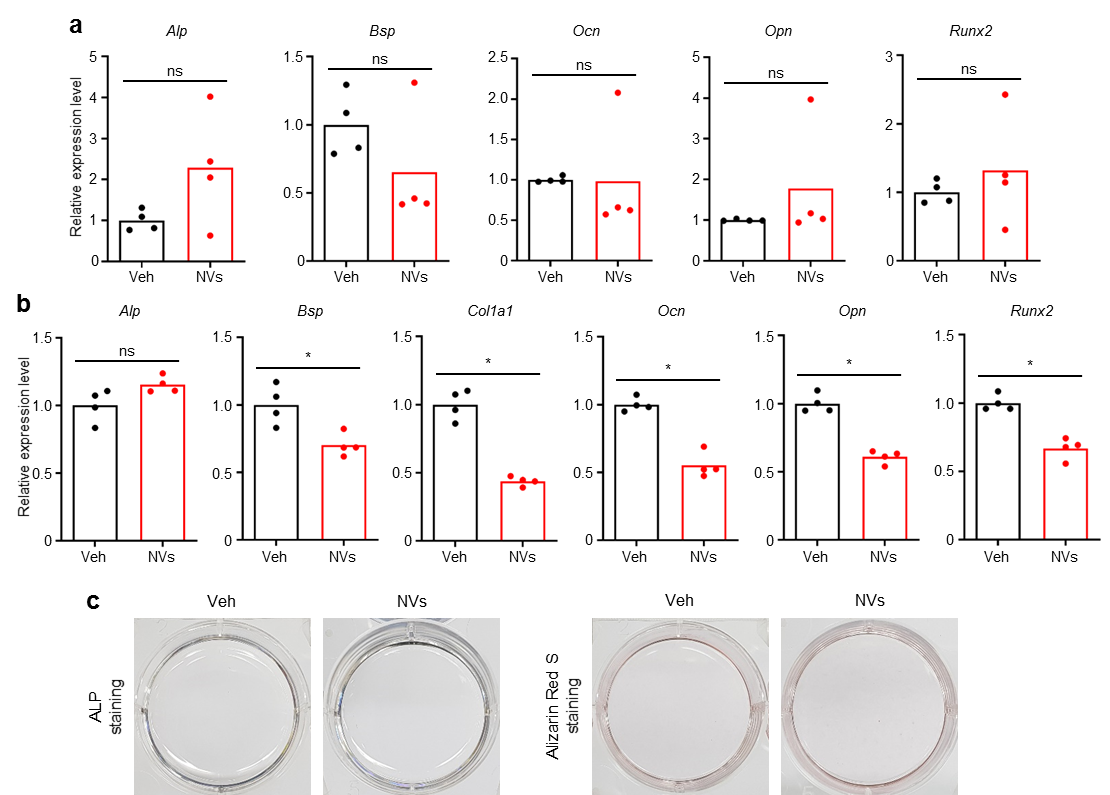

Supplement: Supplementary file 1 [file ijms-21-06425-s001.zip › ijms-901766-supplementary/200731 Figure S1.png]
